# Chronic remote ischemic conditioning for symptomatic internal carotid or middle cerebral artery occlusion: A prospective cohort study

**DOI:** 10.1111/cns.13874

**Published:** 2022-06-15

**Authors:** Sijie Li, Wenbo Zhao, Guiyou Liu, Changhong Ren, Ran Meng, Yuan Wang, Haiqing Song, Qingfeng Ma, Yuchuan Ding, Xunming Ji

**Affiliations:** ^1^ Department of Emergency, Xuanwu Hospital, Beijing Institute for Brain Disorders Capital Medical University Beijing China; ^2^ Beijing Key Laboratory of Hypoxic Conditioning Translational Medicine, Xuanwu Hospital Capital Medical University Beijing China; ^3^ Department of Neurology, Xuanwu Hospital Capital Medical University Beijing China; ^4^ Department of Neurosurgery Wayne State University School of Medicine Detroit MI USA; ^5^ Department of Neurosurgery, Xuanwu Hospital Capital Medical University Beijing China

**Keywords:** impaired hemodynamics, internal carotid artery occlusion, middle cerebral artery occlusion, remote ischemic conditioning

## Abstract

**Aims:**

Remote ischemic conditioning (RIC) has been demonstrated to reduce recurrent stroke in patients with intracranial artery stenosis. This study aimed to evaluate the effects of RIC in patients with the symptomatic internal carotid artery (ICA) or middle cerebral artery (MCA) occlusion.

**Methods:**

This study is based on a high‐volume single‐center prospective cohort study in China, which included patients with symptomatic ICA or MCA occlusion with impaired hemodynamics and receiving chronic RIC. Clinical follow‐up visits were performed regularly, and cardio‐cerebrovascular events were assessed.

**Results:**

In total, 131 patients (68 with ICA occlusion and 63 with MCA occlusion; mean age, 52.6 ± 13.7 years; stroke, 73.5%; transient ischemic attack TIA, 26.5%) qualified for the analysis; the mean follow‐up period was 8.8 years (range, 3–14 years). The compliance of RIC was 95.6 ± 3.7%, and no associated severe adverse events happened. The annual risk of ischemic stroke and ischemic cerebrovascular events was 2.4% and 3.3%, respectively. The cumulative probabilities of ischemic cerebrovascular events and major adverse cardiovascular and cerebrovascular events were 32.8% and 44.8% at 14 years, respectively.

**Conclusion:**

In patients with symptomatic ICA or MCA occlusion with impaired hemodynamics, chronic RIC is well‐tolerated, and it appears to be associated with a low annual risk of ischemic stroke and cardio‐cerebrovascular events.

## Graphical abstract

This study found that in patients with symptomatic cerebral artery occlusion who had impaired hemodynamics, adjunctive therapy with chronic (>10 years) remote ischemic conditioning RIC was well tolerated, and it might be associated with low annual risks of stroke (2.4%) and ischemic cerebrovascular events (3.3%). In addition, the cumulative probabilities of ischemic cerebrovascular events up to 14 years was 32.8%.

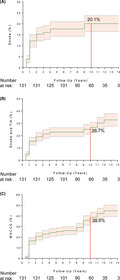

## 1 INTRODUCTION

The internal carotid artery (ICA) is the most common artery involved in atherosclerosis, followed by the middle cerebral artery (MCA).^1^ Occlusion of the ICA and MCA reduces cerebral blood perfusion and increases the risk of ischemic stroke, which accounts for more than one‐fourth of cases of ischemic stroke in the carotid territory.^2^, ^3^, ^4^ Improvement of regional cerebral blood flow (rCBF) has been considered a therapeutic option for patients with intracranial atherosclerosis. However, large trials investigating extracranial–intracranial bypass surgery in patients with symptomatic ICA or MCA atherosclerosis have failed to demonstrate its clinical benefits,^5^, ^6^, ^7^ leading to the standard management of symptomatic ICA or MCA occlusion mainly involving antithrombotic medication and vascular risk factor modification.^8^ However, the annual risk of stroke in patients with symptomatic ICA or MCA occlusion remains to be 5%–6% in general and up to 35.6% for those with decreased rCBF and regional cerebrovascular reactivity to acetazolamide,^9^, ^10^ which causes significant challenges in clinical practice.

Remote ischemic conditioning (RIC) is a systematic protective strategy in which one or more cycles of brief, nonlethal limb ischemia confers protection against prolonged, severe ischemia in distant organs.^11^ Recently, it has been suggested that RIC is a promising strategy in reducing recurrent stroke or transient ischemic attacks (TIAs) in patients with symptomatic intracranial atherosclerosis. RIC also improves cerebral perfusion and promotes arterial collateral opening and reconstruction.^12^, ^13^ As the protective period of one session of RIC persists for approximately 96 h with a 12–24 h unprotected period, adjunctive chronic RIC has been proposed to treat ischemic cerebrovascular diseases and proven safe and effective in this patient population.^14^, ^15^, ^16^ However, it remains unknown whether adjunctive chronic RIC is safe and effective in patients with symptomatic ICA or MCA occlusion.

In this prospective cohort study with long‐term follow‐up (8.8 years), we aimed to assess the safety and potential clinical benefits of chronic RIC as adjunctive therapy for the treatment of symptomatic ICA or MCA occlusion.

## 2 METHODS

### 2.1 Study design and patients

This study was an observational study of a high‐volume single‐center cohort in which a prospectively designed follow‐up schedule was used for the follow‐up of all patients by experienced stroke neurologists. Since 2007, RIC has been used for the treatment of ischemic cerebrovascular disease in Xuanwu Hospital of Capital Medical University, and all patients who received adjunctive RIC were enrolled in this prospective cohort study. Herein, patients aged≥18 years at the first visit who had ischemic stroke or TIA secondary to ICA or MCA occlusion (i.e., occlusion of the extracranial and intracranial segments of the ICA occlusion or M1 segment of the MCA) within 6 months of ictus along with impaired hemodynamics in the territory of the MCA were recruited. Meanwhile, patients diagnosed with moyamoya disease; patients who underwent extracranial–intracranial bypass surgery during follow up; patients with occlusion secondary to non‐atherosclerotic disease, including artery dissection, arteritis, and fibromuscular dysplasia; and patients who received RIC irregularly were excluded. Cerebral hemodynamics were evaluated using single‐photon emission computed tomography (SPECT) and semiquantitative analyses; impaired hemodynamics were defined as an rCBF of the affected side/unaffected side (or the affected side/cerebellum in cases of moderate‐to‐severe contralateral ICA or MCA stenosis or occlusion) of <0.9.

This study was approved by the ethics committee of Xuanwu Hospital of Capital Medical University and was conducted in accordance with the Good Clinical Practice guidelines and Declaration of Helsinki. Written informed consent was obtained from all patients or their legal representatives prior to enrollment in this cohort study.

### 2.2 Interventions

All enrolled patients were managed in accordance with guidelines^17^, ^18^, ^19^ and received the best medical therapies, including modifiable risk factor management, antiplatelet or anticoagulant medication, and statin therapy. Antihypertensive, antidiabetic, and homocysteine‐lowering agents were administered as required, depending on the presence of comorbidities. In addition, all patients received RIC once or twice a day, consisting of five cycles of simultaneous bilateral upper arm ischemia for 5 min, followed by reperfusion for another 5 min. RIC was performed using an electric autocontrol device with cuffs inflated to a pressure of 200 mmHg during the ischemic period and deflated to a pressure of 0 mmHg during the reperfusion period. The procedure was performed by the patients themselves or with the help of their caregivers at home.

All electric autocontrol devices were equipped with subscriber identification module cards carrying patient‐unique identification numbers and recording dates of RIC implementation and connected to a background monitoring platform. To ensure compliance to RIC, the patients were automatically remined by the background monitoring platform when the treatment had been interrupted for four consecutive days, and the investigators were alerted when it had been interrupted for seven consecutive days; all this information was documented in the background monitoring platform.

### 2.3 Follow‐up

All enrolled patients were required to undergo clinical follow‐up visits every 6 months or when necessary in the initial two years, and every 12 months or when necessary thereafter. During the visits, the patients were interviewed regarding recurrent stroke and TIAs, cardiovascular events, symptoms of heart failure, bleeding, new hospitalizations (and their causes), RIC, current medications, control of cerebrovascular disease risk factors, and any other discomfort that they may have complained of. This information was documented in the database of the cohort study.

In addition, a systematic telephone call was conducted for every patient to confirm their medical history between January and February 2021. During the telephone interviews, all patients were also asked regarding recurrences of TIAs and stroke; angina, acute myocardial infarction, or heart failure; new hospitalizations (and causes); bleeding; current medical therapies and RIC; and cerebrovascular risk factor status.

### 2.4 SPECT examination

The cerebral perfusion status was assessed using SPECT (Siemens E. Cam SPECT Workstation, Siemens Healthineers,) prior to enrollment into this study and post RIC treatment. The rCBF was evaluated using technetium‐99 m ethylene cysteine dimer (^99m^Tc‐ECD) and a dual‐headed rotating γ camera at 30 min after intravenous ^99m^Tc‐ECD (25 mCi) bolus injection. Qualitative and semiquantitative analyses of the rCBF were performed using an automated region of interest (ROI) analysis. The ROI was classified according to the territory of the major blood vessels supplying the brain, including the ICA and vertebral arteries. The activity of each signal was compared to the maximal signal uptake in the cerebellum. The heterogeneity of brain perfusion was measured as the coefficient of variation, which was defined as the ratio of the standard deviation to the mean. All SPECT images were evaluated by two independent nuclear medicine physicians.

### 2.5 Data collection

This study included the following patients variables from the database: age, sex, medical history, clinical manifestation of ICA or MCA occlusion, concomitant cerebral artery occlusion or stenosis, combined therapies, baseline cerebral hemodynamics assessed using SPECT, cerebrovascular events, cardiovascular events, and any other clinical events reported by the patients themselves. To ensure accuracy, two independent reviewers matched all patient data with the medical records, and any inconsistent or vague information was clarified after confirmation with the patients.

### 2.6 Outcome assessment

The primary outcome was the proportion of patients who experienced recurrent ischemic cerebrovascular events, including ischemic stroke, TIAs, and amaurosis fugax. The secondary outcomes included the rate of compliance RIC and proportion of patients who experienced cardiovascular events (e.g., angina and myocardial infarction), major adverse cardiovascular and cerebrovascular events (MACCEs), and those who died. The rate of compliance RIC was calculated using the following formula: months of regular RIC/total months of follow‐up; regular RIC was defined as no interruption of RIC or interrupted RIC for ≤4 consecutive days. MACCEs were defined as all‐cause death, stroke or TIAs, acute coronary syndrome (e.g., myocardial infarction and angina), and heart failure requiring hospitalization.

### 2.7 Statistical analysis

Categorical variables were reported as *n* (%) and continuous variables as means ± standard deviations or medians [25th to 75th interquartile ranges (IQRs)] depending on the variable distribution. Survival curves for time‐to‐event variables were obtained using Kaplan–Meier estimates. All data were analyzed using SPSS (version 24.0; IBM Inc.) with the significance level set al *p* < 0.05. Kaplan–Meier curves were constructed using Prism (version 6.02; GraphPad Software Inc.).

## 3 RESULTS

Between January 2007 and December 2017, a total of 664 patients with ICA or MCA occlusion were treated with adjunctive RIC. Of these patients, 376 did not have impaired hemodynamics or were asymptomatic; 69 were diagnosed with moyamoya disease; 57 underwent extracranial–intracranial bypass surgery during the study period; 12 had occlusion secondary to non‐atherosclerotic disease; and 19 underwent RIC irregularly. Therefore, a total of 131 patients finally were recruited to this study, including 68 patients with symptomatic ICA occlusion and 63 patients with symptomatic MCA occlusion.

### 3.1 Baseline characteristics

The baseline characteristics of all recruited patients are summarized in Table 1. Their mean age was 52.6 ± 13.7 years, and 84 patients (64.1%) were men. The median time interval from symptom onset to RIC was 65 (IQR, 46–85) days. Ninety‐seven patients (74.0%) had ischemic stroke, while 34 patients (26.0%) had TIAs. Twenty‐five patients (19.1%) had concomitant intracerebral arterial stenosis (>50%), and 17 patients (13.0%) had concomitant intracerebral arterial occlusion. The rate of compliance to RIC was 95.6 ± 3.7%, and no severe adverse events were associated with RIC.

**TABLE 1:** Baseline Characteristics of the Study Population

	*N* = 131
Age, years, mean ± SD	52.6 ± 13.7
Male, *n* (%)	84 (64.1)
Clinical manifestations, *n* (%)	
Ischemic stroke	97 (74.0)
TIA	34 (26.0)
Interval from symptom onset to RIC treatment, day, median (IQR)	65 (46–85)
Interval from symptom onset to SPECT examination, day, median (IQR)	50 (37–69)
Concomitant cerebral artery stenosis, *n* (%)	25 (19.1)
Concomitant cerebral artery occlusion, *n* (%)	17 (13.0)
Risks of cerebrovascular disease, *n* (%)	
Hypertension	79 (60.3)
Diabetes mellitus	41 (31.3)
Dyslipidemia	61 (46.6)
Previous stroke	35 (26.7)
Smoking	41 (31.3)
Drinking	30 (22.9)
Coronary artery disease	16 (12.2)
Hyperhomocysteinemia	26 (19.8)
HDL cholesterol level, mean ± SD, mmol/L	1.2 ± 0.3
LDL cholesterol, mean ± SD, mmol/L	2.5 ± 1.0
Triglycerides, mean ± SD, mmol/L	1.5 ± 0.8
Glucose, mean ± SD, mmol/L	6.0 ± 1.9
Homocysteine, median (IQR), mmol/L	13.1 (10.6–16.1)
Hemoglobin level, mean ± SD, mmol/L	144.5 ± 25.9
Fibrinogen level, mean ± SD, g/L	3.5 ± 0.7
Hs‐CRP, median (IQR), mmol/L	0.6 (0.3–1.7)
Compliance of RIC treatment, %	95.6 ± 3.7

Abbreviations: HDL, high density lipoprotein; Hs‐CRP, hypersensitive C‐reactive protein; IQR, interquartile range; LDL, Low Density Lipoprotein; RIC, remote ischemic conditioning; SD, standard deviation; SPECT, single‐photon emission computed tomography; TIA, transient ischemic attack.

Among the 131 patients, 79 (60.3%) had hypertension; 41 (31.3%), diabetes mellitus; 61 (46.6%), dyslipidemia; and 16 (12.2%); coronary artery disease. At the time of enrollment, the mean level of low‐density lipoprotein cholesterol was 2.5 ± 1.0 mmol/L; mean level of triglyceride, 1.5 ± 0.8 mmol/L; mean level of fasting glucose, 6.0 ± 1.9 mmol/L; median level of homocysteine was 13.1 (IQR, 10.6–16.1) mmol/L, and median level of hypersensitive C‐reactive protein, 0.6 (IQR, 0.3–1.7) mmol/L. The ther risk factors of cerebrovascular disease and baseline hemoglobin and fibrinogen levels are shown in Table 1.

### 3.2 Cerebrovascular risk factor control

The medical therapies and cerebrovascular risk factor control status at the latest follow‐up visit are summarized in Table 2. The mean follow‐up period was 8.8 (range, 3–14) years. At the latest follow‐up visit, 116 patients (88.5%) received antithrombotic medications, and 119 patients (90.8%) did not smoke cigarettes. The systolic blood pressure in 82 patients (62.6%) was lower than 140 mmHg, and the diastolic blood pressure in 110 patients (84.0%) was lower than 90 mmHg. The mean level of low‐density lipoprotein cholesterol was 2.4 ± 0.9 mmol/L; mean level of triglycerides, 1.4 ± 0.7 mmol/L; mean level of fasting glucose was 6.0 ± 1.5 mmol/L, mean level of homocysteine was 12.1 ± 4.0 mmol/L, and median level of hypersensitive C‐reactive protein, 0.5 (IQR, 0.3–1.2) mmol/L.

**TABLE 2:** Medical Therapy and Cerebrovascular Risk Factor Status at Latest Follow‐up

	*N* = 131
Follow‐up, years, mean (range)	8.8 (3–14)
Use of antithrombotic medication	116 (88.5)
Systolic blood pressure ≤ 140 mmHg	82 (62.6)
Diastolic blood pressure ≤ 90 mmHg	110 (84.0)
Not currently smoking cigarettes, *n* (%)	119 (90.8)
LDL cholesterol, mean ± SD, mmol/L	2.4 ± 0.9
Triglycerides, mean ± SD, mmol/L	1.4 ± 0.7
Glucose, mean ± SD, mmol/L	6.0 ± 1.5
Homocysteine, mean ± SD, mmol/L	12.1 ± 4.0
Hs‐CRP, median (IQR), mmol/L	0.5 (0.3–1.2)

*Note:* Values of data are from the last follow up.

Abbreviations: Hs‐CRP, hypersensitive C‐reactive protein; IQR, interquartile range; LDL, Low Density Lipoprotein; SD, standard deviation.

### 3.3 Clinical events

The clinical events during follow‐up are summarized in Table 3. A total of 28 patients (21.4%) experienced ischemic stroke, while eight patients experienced TIAs. The annual risk of ischemic stroke was 2.4%, and that of combined ischemic stroke and TIAs was 3.3%. Six patients (4.6%) died: One died from a gastrointestinal tumor, one from leucocythemia, two from pneumonia, and the remaining two from acute myocardial infarction. Six patients (4.6%) experienced acute coronary syndrome. In total, 46 patients (35.1%) experienced MACCEs during the study period. In addition, a female patient was diagnosed with lung adenocarcinoma in the fourth year of treatment; a male patient was diagnosed with gastric cancer in the fifth year of treatment; one patient was diagnosed with vascular cognitive impairment in the third year of treatment; three patients experienced intracerebral hemorrhage; and two patients experienced gastrointestinal hemorrhage.

**TABLE 3:** Clinical Events During Follow‐up

	*N* = 131
Death, *n* (%)	6 (4.6)
Stroke, *n* (%)	28 (21.4)
Composite of stroke or TIA, *n* (%)	36 (27.5)
Acute cardiovascular events, *n* (%)	6 (4.6)
MACCE^†^, *n* (%)	46 (35.1)

Abbreviations: MACCE, major adverse cardiac and cerebral events; TIA, transient ischemic attack.

^†^MACCE was defined as all‐cause mortality, stroke or transient ischemic attack, nonfatal MI, acute coronary syndrome (including unstable angina), and left ventricular failure requiring hospital admission.

**FIGURE 1:**
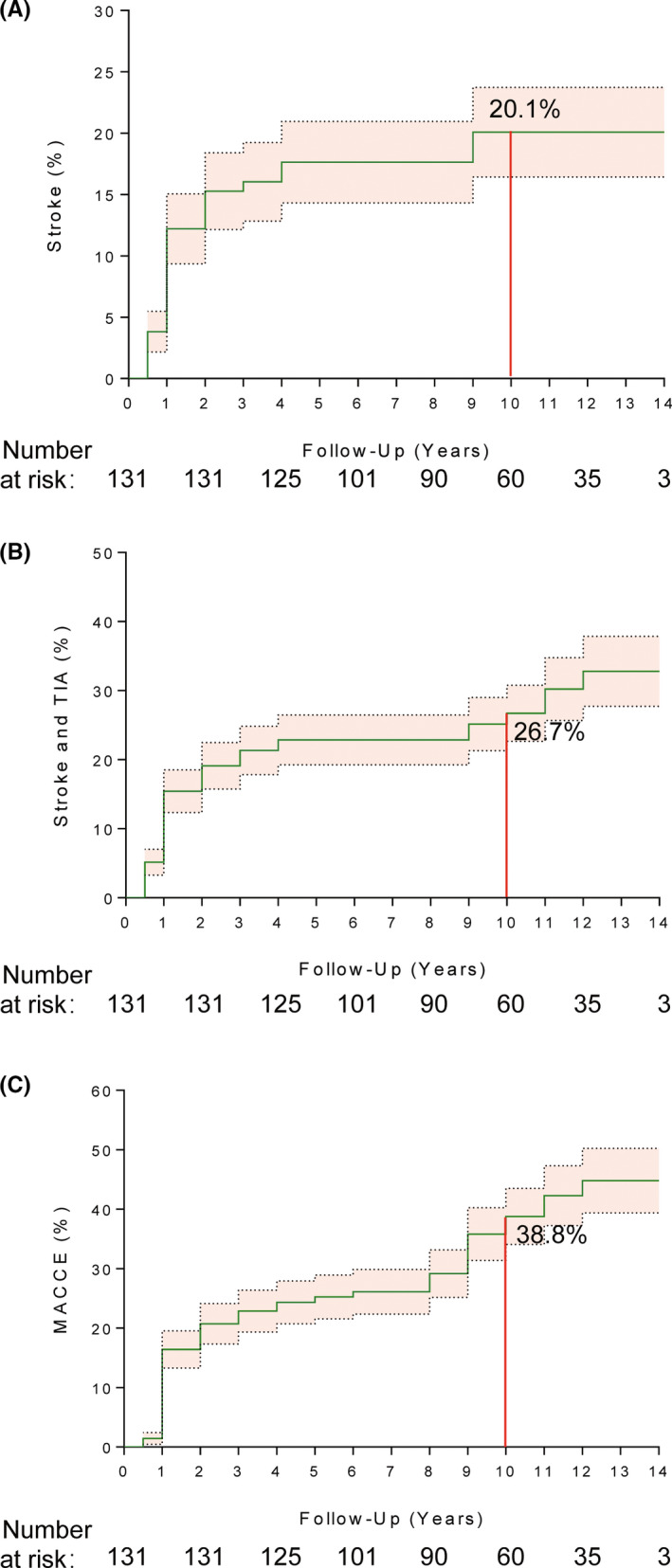
Kaplan–Meier estimates for clinical events at 14‐year follow‐up. Kaplan–Meier plot showing cumulative probability of ischemic stroke (A); ischemic stroke and transient ischemic attack (TIA) (B); major adverse cardiovascular and cerebrovascular events (MACCE) (C)

The Kaplan–Meier curves for recurrent stroke, composite stroke and TIAs, and MACCEs with 14 years of RIC are illustrated in Figure 1. The survival analysis indicated that the cumulative probabilities of stroke were 12.2%, 16.0%, 17.6%, and 20.1% after 1, 3, 5, and 10 years, respectively; the cumulative probabilities of composite stroke and TIAs were 15.4%, 21.3%, 22.9%, 26.7%, and 32.8% at 1, 3, 5, 10, and 14 years, respectively; and the cumulative probabilities of MACCEs were 16.4%, 22.9%, 25.2%, 38.8%, and 44.8% at 1, 3, 5, 10, and 14 years, respectively.

## 4 DISCUSSION

In this study, patients with symptomatic ICA or MCA occlusion who had impaired hemodynamics and received chronic (>10 years) RIC as adjunctive therapy exhibited lower annual risks of stroke (2.4%) and ischemic cerebrovascular events (3.3%). In addition, rates of 4.6% for mortality and 4.6% for the occurrence of cardiovascular events were observed during the follow‐up period, and the cumulative probabilities of ischemic cerebrovascular events and MACCEs up to 14 years were 32.8% and 44.8%, respectively.

During the long‐term follow‐up, the proportion of patients who smoked and level of homocysteine significantly decreased; however, the low‐density lipoprotein cholesterol and fasting glucose levels remained poorly controlled, which is detrimental to the prevention of cerebrovascular disease. In addition, the proportion of patients whose systolic blood pressure was controlled at lower than 140 mmHg was less than two‐thirds. Although all recruited patients were required to undergo regular clinical visits, and patient education on stroke prevention was implemented during these clinical visits, the control of cardiovascular risk factors remained unsatisfactory, which may partly reflect the suboptimal control of cardiovascular risk factors in clinical practice.^20^, ^21^ Even with the poor control of these conditions, the results of this study revealed low odds of ischemic cerebrovascular events, mortality, and MACCEs in patients with symptomatic ICA or MCA occlusion with such a long‐term follow‐up.

Previous studies have demonstrated that among with ICA or MCA occlusion, the annual risk of stroke is 12.5% in those with any impaired hemodynamics, and is much higher at 35.6%–41.4% in patients with severely impaired hemodynamics, the annual risk of stroke.^22^, ^23^, ^24^ In our study, we recruited patients with symptomatic ICA or MCA occlusion who had impaired hemodynamics, as measured using SPECT, and the annual risk of all‐cause stroke was 2.4%, which was notably lower than previously reported. The discrepancy in the annual risk of stroke between this study and previous studies may have been attributable to several reasons. First, chronic RIC is associated with an improved cerebral perfusion status in the ischemic territory, as shown by the SPECT results, and an improved cerebral tissue tolerance to ischemic injury, both of which may improve brain tissue tolerance to ischemic insults. Second, the modification of risk factors of cerebrovascular diseases and antiplatelet therapy have been advancing greatly over the past few decades, which might have also contributed to the low risk of recurrent ischemic cerebrovascular events. In addition, stroke is more likely to recur within the first few months after ischemic stroke. In this study, the median time interval from symptom onset to enrollment was approximately 2 months, which might have led to missing the high stage of recurrent stroke.

In previous studies that investigated RIC in patients with moyamoya disease and symptomatic intracranial atherosclerosis, RIC was found to reduce ischemic cerebrovascular events and improve the cerebral perfusion status, as evaluated using SPECT or positron emission tomography.^12^, ^25^ However, these studies mainly included patients with cerebrovascular stenosis, and the follow‐up period for most patients was less than 2 years. To the best of our knowledge, this study is the first to reveal the potential effects of RIC in patients with symptomatically atherosclerotic ICA or MCA occlusion with long‐term follow up. Consistent with previous studies that investigated RIC in patients with atherosclerotic intracranial artery stenosis, this study also found that RIC was associated with reduced ischemic cerebrovascular events in patients with major cerebral arterial occlusion, which may further extend the scope of the protective effects of RIC. Recently, several non‐invasive modalities for long‐term CBF monitoring in humans have been reported,^26^, ^27^, ^28^ and these modalities help facilitate an easier evaluation of the cerebral blood flow and improve the quality of future studies investigating RIC in patients with arterial occlusion or stenosis.

As a physical therapy, RIC must be performed for 45 min or more daily, which might cause tissue injury and discomfort, and thus influence the compliance of patients. Intriguingly, this study found that the compliance of patients was high, which may have been attributed to the timely automatic reminder of the cloud platform. Furthermore, all the patients tolerated the procedure with few complaints of pain. This may confirm that pain tolerance can also be adapted, which is in line with the findings of previous studies that showed that 20 min of painful stimulation once daily for eight consecutive days may substantially decrease pain ratings in response to a constant pain stimulus^29^ and that the pain threshold gradually increases in the ischemic arm over the course of each cycle of RIC.^30^

This study had limitations. First, this study has a prospective single‐center cohort study design, whose inherent limitation is related to certain biases. However, it is difficult to conduct a randomized controlled trial with such a long‐term follow‐up, and a long‐term cohort study might be a better substitute. Second, the number of patients in this study was relatively small, which might have influenced the study results. Therefore, the findings should be further verified in larger trials and interpreted with caution. Furthermore, considering the long‐term follow‐up, the number of patients lost to follow‐up was relatively small; however, the possibility of missing adverse events cannot be completely ruled out, especially considering the incidence of TIA and amaurosis fugax related to the inherent characteristics of the disease.

In conclusion, chronic RIC is well tolerated with high compliance and may be associated with low rates of ischemic cerebrovascular events and MACCEs in long‐term follow‐ups among patients with symptomatic ICA or MCA occlusion with impaired hemodynamics. As it is difficult to conduct randomized controlled trials investigating the effects of chronic RIC in patients with symptomatic ICA or MCA occlusion with such a long‐term follow‐up, larger cohort studies are needed to confirm the results, so that a meta‐analysis may be conducted to clarify the overall clinical benefits of chronic RIC in this patient population.

## AUTHOR CONTRIBUTIONS

Dr. Sijie Li, Wenbo Zhao, and Xunming Ji contributed to the conception and design of the study. Dr. Guiyou Liu, Changhong Ren, Ran Meng, Yuan Wang, Haiqing Song, and Qingfeng Ma contributed to the acquisition and analysis of the data. Dr. Sijie Li, Wenbo Zhao, Yuchuan Ding, and Xunming Ji contributed to drafting and revising the manuscript, and preparing the figures.

## CONFLICT OF INTEREST

Dr. Xunming Ji is one of the inventors of the electric autocontrol device that has been patented in China. The other authors report no disclosures.

## Data Availability

The data, analytic methods, and study materials of this study are available from the corresponding author on reasonable request and detailed protocol is provide.
